# Hierarchical CdMoO_4_ nanowire–graphene composite for photocatalytic hydrogen generation under natural sunlight†

**DOI:** 10.1039/c8ra01557k

**Published:** 2018-04-12

**Authors:** Sunil R. Kadam, Rajendra P. Panmand, Shashikant Tekale, Supriya Khore, Chiaki Terashima, Suresh W. Gosavi, Akira Fujishima, Bharat B. Kale

**Affiliations:** Centre for Advanced Studies in Materials Science, Department of Physics, Savitribai Phule Pune University, (Formerly University of Pune) Ganeshkhind Pune-411007 India; Centre for Materials for Electronics Technology (C-MET), Ministry of Electronics and Information Technology (MeitY), Government of India Panchawati, Off. Pashan Road Pune-411008 India bbkale@cmet.gov.in; Photocatalysis International Research Center, Research Institute for Science & Technology, Tokyo University of Science 2641 Yamazaki, Noda Chiba 278-8510 Japan

## Abstract

Herein, a facile *in situ* solvothermal technique for the synthesis of a CdMoO_4_/graphene composite photocatalyst is reported. Graphene oxide (GO) was synthesised by an improved Hummers' method and was further used for the *in situ* synthesis of graphene *via* GO reduction and the formation of a CdMoO_4_ nanowire/graphene composite. The structural phase formation of tetragonal CdMoO_4_ was confirmed from X-ray diffraction measurements. The small nanoparticle assembled nanowires, prismatic microsphere morphology and crystalline nature of the synthesized material were investigated using field emission scanning electron microscopy (FESEM) and transmission electron microscopy (TEM). Due to its unique morphology and stability, the CdMoO_4_/graphene composite was used as a photocatalyst for H_2_O splitting. In comparison to pristine CdMoO_4_, the CdMoO_4_/graphene composite showed the best hydrogen evolution rate, *i.e.* 3624 μmole h^−1^ g^−1^, with an apparent quantum yield of 30.5%. The CdMoO_4_/graphene composite has a higher photocatalytic activity due to the inhibition of charge carrier recombination. H_2_ production measurements showed that the ternary semiconductor/graphene composite has enhanced photocatalytic activity for H_2_ generation.

## Abstract 2

Herein, a facile *in situ* solvothermal technique for the synthesis of a CdMoO_4_/graphene composite photocatalyst for hydrogen generation under natural solar light.

## Graphical abstract

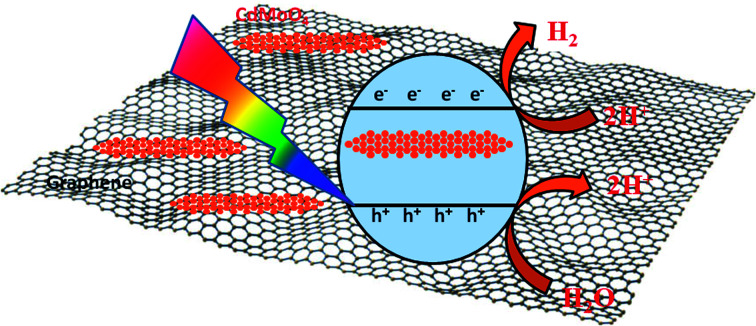

## 1. Introduction

Hydrogen has been considered as one of the best clean energy fuels owing to its green and renewable properties, compared with those of other fuels (such as methane, coal, gasoline, *etc.*), which could be used to power electronic devices, homes and automobiles.^1,2^ Conservative energy resources have been depleted to a large extent because of continuously rising energy demands.^3^ Therefore, it is essential to generate alternative eco-friendly and economically viable fuels, which can fulfil both current and future energy requirements. Hydrogen has been identified as a potential eco-friendly energy carrier in many low greenhouse gas energy scenarios.^4^ However, Arbuj *et al.* have previously discussed that only limited success has been achieved in the generation and storage of hydrogen.^4^ The different efficient ways to produce hydrogen, using, for example, photocatalytic, electrochemical and photoelectrochemical methods, have attracted much attention.^1,5,6^ Currently, coal gasification and the steam reformation of methane are the well-known and promising methods for high purity hydrogen production, but these are energy intensive processes and are undesirable in terms of the effect that they have on the environment.^7,8^ However, the technology to produce hydrogen in a cost-effective and eco-friendly way has not yet been developed. Considering the overall scenario and the abundant water supply on the Earth, hydrogen generation *via* photocatalytic water spitting is the most promising technique for renewable hydrogen production.^9^

The first electrochemical generation of hydrogen catalyzed by a TiO_2_ photoanode with platinum as the counter electrode in saturated aqueous electrolyte was reported by Fujishima and Honda *et al.*^10,11^ In the literature, various semiconductor oxides such as TiO_2_,^12,13^ ZnO,^5^ SnO_2_/SnS_2_,^14–16^ WO_3_,^17^ Ga_2_O_3_,^18^ and ZnS,^9^ have been reported to be good photocatalysts for energy, as well as environmental related applications. However, most of these binary oxide photocatalysts have wide band gaps and show better activity in the presence of UV light, which only accounts for 5% of the solar spectrum, and do not show good photocatalytic activity in visible light.^19^ Furthermore, there have been attempts to develop anion doped TiO_2_,^20,21^ ZnO^5^ nanomaterials and Fe_2_O_3_,^22^ Bi_2_O_3_,^23^ BiVO_4_,^17^ MoO_3_,^1^*etc.* as visible light active photocatalysts for H_2_ generation. However, the stability of these materials limits their commercial use as active photocatalysts. Hence, researchers have focused their attention on the development of stable and efficient ternary semiconductor photocatalysts for H_2_ production.^24^

Molybdenum based semiconductor catalysts have gained increasing attention because of their potential technological importance for a wide range of applications, such as photocatalytic hydrogen generation,^25,26^ environmental pollutant dye degradation,^27,28^ and in high density Li-ion batteries.^29^ In this context, we have attempted to develop the synthesis of ternary CdMoO_4_*via* a simple solvothermal method. In the literature, there are some reports on the synthesis of CdMoO_4_,^30–32^ CdMoO_4_–graphene,^33^ Ag–CdMoO_4_ (ref. 34) and studies on their photocatalytic dye degradation, but there have been no reports on photocatalytic hydrogen generation using these materials. Enormous efforts have been made on the decoration of noble metals, such as Pt, on catalyst surfaces in order to increase the efficiency of photocatalysts. However, scaling up platinum-based catalysts is questionable due to the scarcity of Pt in the Earth's crust.^35^ Graphene is a well-known and significant material due to its two-dimensional layered sheet-like structure, which results in its remarkable properties. Graphene, in its composite form with semiconductor materials, has exhibited comparatively good photocatalytic activity over individual semiconductors due to its outstanding properties, such as high electron mobility, large surface area, excellent conductivity, stability and mechanical strength.^36–38^ In a graphene composite material, the 2D layered conjugated structure of graphene helps to reduce the electron and hole pair recombination time, which is beneficial for photocatalytic activity. During photocatalysis experiments, these carbon-related materials will act as both an electron-acceptor and an electron-transport material, facilitating the migration of photoinduced electrons and obstructing charge recombination to enhance the photocatalytic performance.^39^ To date, there have been reports on graphene and semiconductor oxide/sulfide composite photocatalysts, such as N–TiO_2_/graphene,^40^ CeMoO_4_,^41^ MoS_2_–graphene,^42^ BiFeO_3_–graphene,^43^ ZnO–graphene^44^ and Bi_2_WO_6_ (ref. 45) for energy and environmental related applications in the presence of UV/Visible light. Considering the significant characteristics of graphene, it is a potential support material for stabilizing the CdMoO_4_ photocatalyst, which enhances hydrogen generation by photocatalytic water splitting. The interfacial contact between the CdMoO_4_/graphene composite is responsible for its significant photocatalytic activity. The present study reports the *in situ* synthesis of the CdMoO_4_ nanostructure on few layered graphene *via* a solvothermal method. The structural and optical properties of the resulting CdMoO_4_/graphene composite materials have been thoroughly studied. The synthesized materials were used as photocatalysts for enhanced hydrogen generation *via* water splitting under solar light irradiation.

## 2. Experimental methods

### 2.1 Synthesis of graphene and its composites with CdMoO_4_ nanowires

All of the chemicals used for the synthesis of graphene oxide (GO) and CdMoO_4_ were analytical reagent grade and used without any further purification. The synthesis of GO was carried out using a previously reported improved Hummers' method.^46^ In the GO synthesis process, concentrated H_2_SO_4_ (69 ml) was added to a mixture of graphite flakes (3.0 g, 1 wt equiv.) containing NaNO_3_ (1.5 g, 0.5 wt equiv.), and the reaction mixture was cooled to 0 °C. Further to this, the addition of KMnO_4_ (9.0 g, 3 wt equiv.) was carried out by keeping the reaction temperature below 20 °C. The reaction mixture was warmed to 35 °C and allowed to stir for 30 min, followed by the slow addition of water (138 ml), producing a large exothermic increase in the reaction temperature to 98 °C. After that, external heating was introduced to maintain the reaction temperature at 98 °C for 15 min, then the heating was stopped, and the reaction was cooled in a water bath for 10 min. Additional water (420 ml) and 30% H_2_O_2_ (3 ml) were added, which produced another exothermic reaction, and the solution was then allowed to cool naturally. After natural cooling, the mixture was centrifuged (6000 rpm for 1 h), and the supernatant was decanted away. The obtained solid was dried overnight at room temperature, resulting in 1.2 g of solid product.

The synthesized GO was used for the further synthesis of graphene/CdMoO_4_ nanowire composites. The detailed method for the synthesis of CdMoO_4_ nanowires and CdMoO_4_ prisms can be found in the ESI† (Experimental work A and B). As per the requirement, graphene oxide was put into a beaker containing methanol : ethylene glycol in a ratio of 3 : 1, denoted as MEG (3 : 1), and kept in an ultrasonic bath for 15 minutes in order to separate the graphene layers. This solution was introduced into a reaction mixture of Cd and Mo precursors, and the resulting solution was transferred into a Teflon lined reactor, which was then packed into a stainless steel jacket, and kept in an oven at 150 °C for 24 hours. After completion of the reaction, the reactor was allowed to cool naturally. The product was filtered using Whatman filter paper no. 41. The product was dried at 80 °C for 4 hours in an oven. The collected powdered sample was further analyzed using various characterization techniques.

### 2.2 Material characterization

The crystalline phases were investigated using powder X-ray diffraction (PXRD, XRD-D8, Advance, Bruker-AXS). The morphologies of the synthesized samples were investigated by field emission scanning electron microscopy (FESEM, Hitachi, S-4800). The optical properties of the powder samples were studied using a UV-visible-near infrared spectrometer (UV-VIS-NIR, PerkinElmer Lambda-950). For HRTEM (JEOL, 2010F instrument) studies, samples were prepared by dispersing the powder in ethanol, followed by ultrasonication in an ultrasonic bath for 5 min. The resulting dispersion was then drop-casted onto a carbon coated copper grid and was subsequently dried under vacuum. Room temperature Raman spectroscopy measurements were performed using a Renishaw InVia microscope Raman system with a laser wavelength of 532 nm in back scattering geometry laser power mode on a 5 mW sample with a laser spot size of 1 μm. The purity of the collected gas was analyzed by gas chromatography (Model Schimadzu GC-14B, MS-5 Å column, TCD, Ar carrier).

### 2.3 Hydrogen generation *via* water splitting

Photocatalytic hydrogen (H_2_) generation *via* water splitting using CdMoO_4_ and its graphene composites as photocatalysts, in an aqueous solution containing methanol as a hole scavenger, was performed in an air tight quartz reactor (with a total reactor volume of 70 ml). The assembly was arranged on a terrace and the quartz reactor containing the reaction mixture was irradiated with solar light. The quartz reactor was equipped with a water circulator in order to absorb the IR radiation, which minimizes the effects of overheating. The intensity of the solar light was measured using a digital Lux meter. The observed average intensity of sunlight reaching the surface of the Earth is 125 000 lux. Here, a known quantity (15 mg) of powder photocatalyst was suspended in a solution containing 20% (v/v) aqueous methanol. The total volume of the reaction mixture was 25 ml and there was 45 ml of free space in the reactor. The reactor was made air tight with a rubber septum and was sonicated for 5 min and then stirred for 30 min to homogenize the suspension. Finally, it was bubbled with ultra-high purity (UHP) nitrogen (N_2_) gas to remove all dissolved gases and purging was continued for 30 min to ensure and inert atmosphere in the reactor. After purging the gas, the flow of N_2_ was stopped and the reactor was made air tight. As soon as the reactor was irradiated with solar light, the photocatalytic splitting of water was initiated and the generated H_2_ gas was collected in the empty head space of the reactor. The amount and purity of the gas evolved was analyzed by gas chromatography (Model Schimadzu GC-14B, MS-5 Å column, TCD, and a porapak-Q packed column under N_2_ as the carrier gas). The apparent quantum efficiency (AQE) of the hydrogen generated was calculated using the following equation:
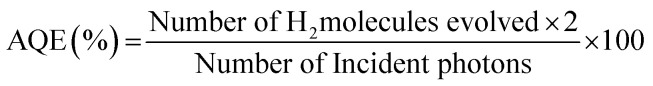


## 3. Results and discussion

**Fig. 1:**
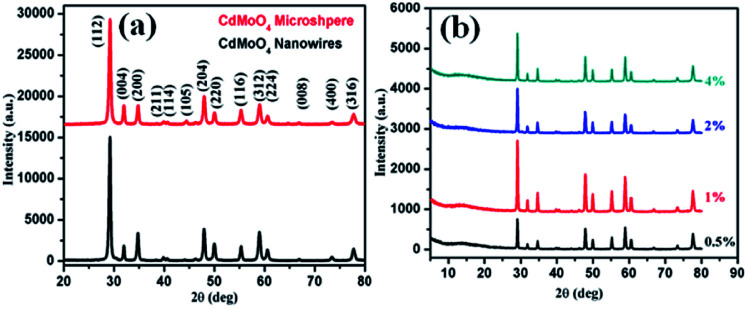
(a) PXRD pattern of CdMoO_4_ synthesized in MEG solvent (3 : 1 and 1 : 1). (b) PXRD of the CdMoO_4_ composites synthesized in MEG solvent (3 : 1) with 0.5%, 1%, 2% and 4% graphene.

The synthesized CdMoO_4_ in a methanol : ethylene glycol ratio of 3 : 1, denoted as MEG (3 : 1), and in a 1 : 1 ratio, denoted as MEG (1 : 1), and the 0.5%, 1%, 2% and 4% graphene composites synthesized using CdMoO_4_ in MEG (3 : 1) were characterized using PXRD, as shown in Fig. 1a and b. The PXRD patterns of CdMoO_4_ synthesized in MEG (3 : 1) and MEG (1 : 1) shown in Fig. 1a show a tetragonal phase formation. The diffraction peaks of CdMoO_4_ synthesized in both solvent systems could be well indexed and show body centred tetragonal phase formation, in good agreement with the reported JCPDS card no. 007-0209. The PXRD peak intensity of the CdMoO_4_ synthesized in MEG (3 : 1) was observed to be slightly higher than that of CdMoO_4_ synthesized in MEG (1 : 1). The peak intensity can be attributed to the crystalline nature and small particle size of the material. Along with the PXRD pattern of the CdMoO_4_ synthesized in MEG (3 : 1), the PXRD patterns of its 0.5%, 1%, 2% and 4% graphene composites are shown in Fig. 1b. The PXRD patterns clearly show that the intensity of the graphene peak at 2*θ* = 14.1 slightly increases upon an increase in the percentage of graphene. In Fig. 1b, in the graphene/CdMoO_4_ composite patterns, there are no extra peaks present, revealing the phase purity of the synthesized samples.

**Fig. 2:**
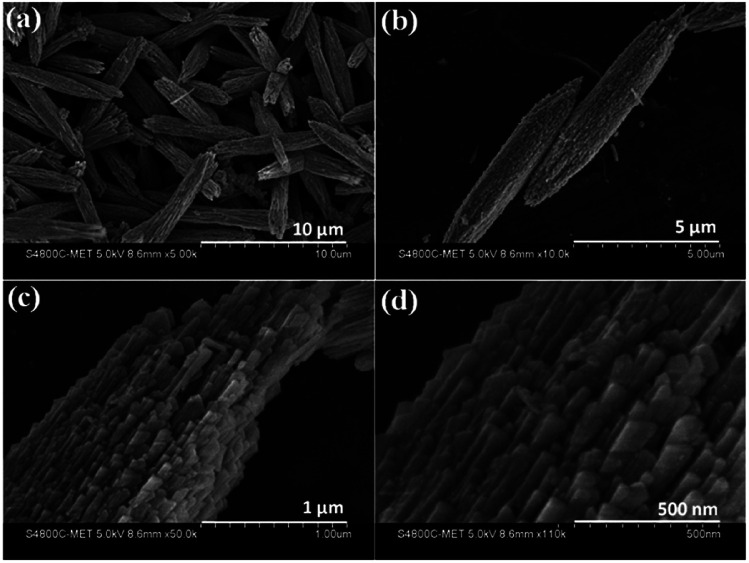
(a–d) FESEM images of as-synthesized CdMoO_4_ in the MEG (3 : 1) solvent system.

Morphological investigations on the as-synthesized CdMoO_4_ in the MEG (3 : 1) solvent system were performed using FESEM and the results are shown in Fig. 2a–d. From the FESEM images, the formation of a nanowire bundle-like morphology can be clearly observed. It is worth noting that a unique nanowire structure of CdMoO_4_ such as this has not been previously synthesized using a solvothermal process. The average size of the CdMoO_4_ nanowire bundles is observed to be 5 μm (Fig. 2a and b). Interestingly, the formation of the nanowire bundle-like structure was achieved through the assembly of nanowires, as observed in Fig. 2b. The average size of the individual nanoparticles was observed to be ∼30 nm in size (Fig. 2c and d). Overall, the FESEM observations show that there is a uniform distribution of the structural morphology.

**Fig. 3:**
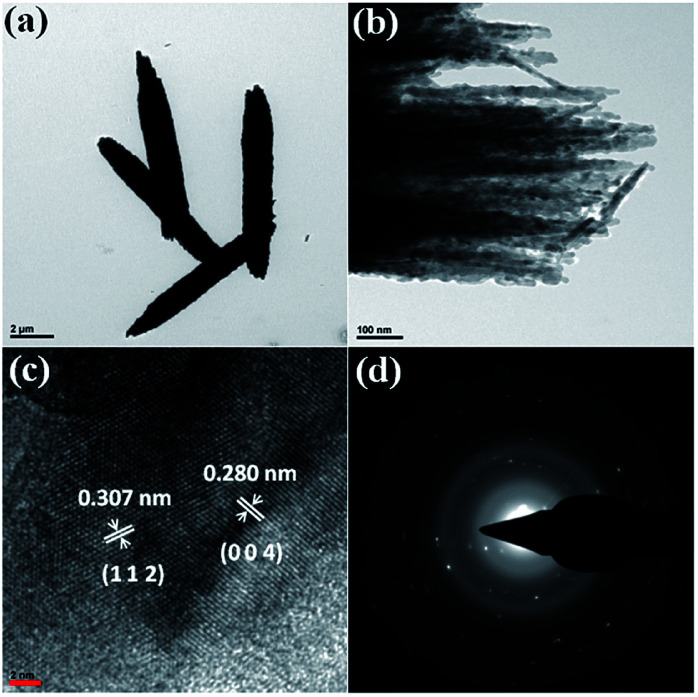
(a and b) TEM images of as-synthesized CdMoO_4_ nanowires. (c) The high-resolution image, and (d) the SAED pattern.

Further, a TEM investigation was performed in order to determine the morphology and crystalline nature of the CdMoO_4_ nanowires (Fig. 3a–d). A long nanowire bundle-like structure was noticed in the TEM images with an average size of 5 μm in length and 1.5 μm in thickness. It was observed that the corresponding nanowire formation takes place *via* the assembly of ordered uniform tiny nanoparticles of ∼20 nm in size (Fig. 3b), which is also in good agreement with the FESEM analysis. Fig. 3c shows the high-resolution (HR) TEM images taken from the edge of a nanoparticle, which provides more detailed structural information. The lattice spacings were observed to be 0.307 nm and 0.280 nm, which correspond to the (112) and (004) planes of tetragonal CdMoO_4_, respectively (Fig. 3c), and are also consistent with the results of the PXRD. Fig. 3d shows the selected area electron diffraction (SAED) pattern of the CdMoO_4_ sample, which clearly shows that it is crystalline in nature.

**Fig. 4:**
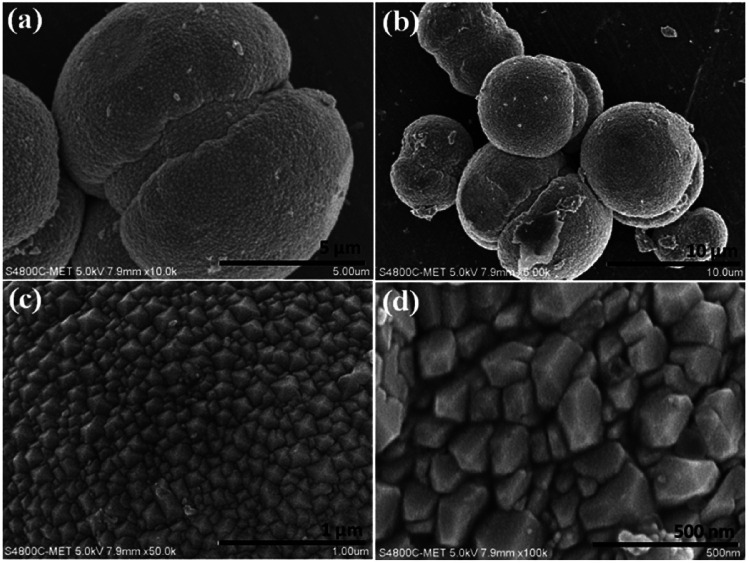
(a–d) FESEM images of as-synthesized CdMoO_4_ in the MEG (1 : 1) solvent system.

Morphological investigations on as-synthesized CdMoO_4_ synthesized in the MEG (1 : 1) system were carried out using FESEM and the results are shown in Fig. 4a–d. From the FESEM images, the formation of a microsphere (5–10 μm)-like morphology can be observed *via* the assembly of tiny close-packed nano prisms. It is also worth noting that such unique microspheres of CdMoO_4_ synthesized *via* a solvothermal process has not been previously observed. More significantly, the nanoparticles at the surface of the structure are prismatic in shape and the overall average size of the nano prisms is 50–100 nm (Fig. 4c and d). Overall, the FESEM observations show that there is a uniform distribution of the microsphere-like morphology.

**Fig. 5:**
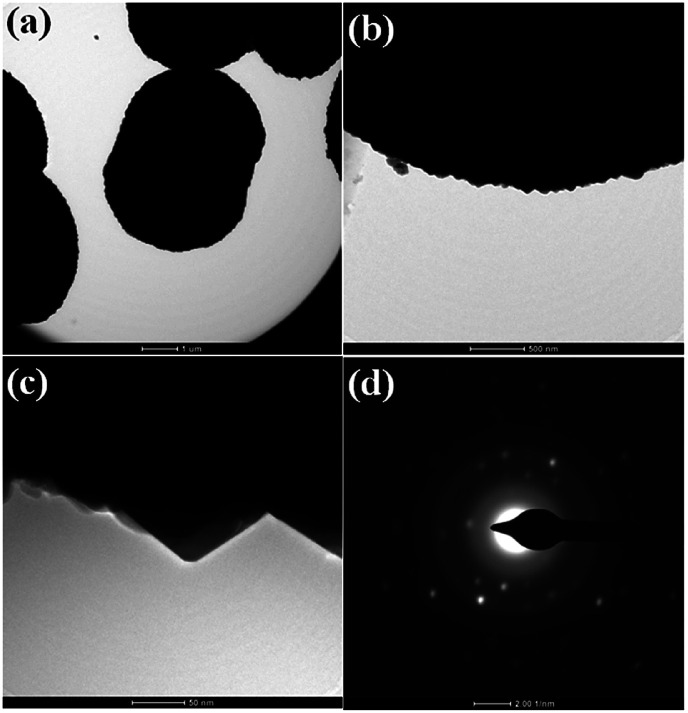
(a and b) TEM images of as synthesized CdMoO_4_ microsphere (c) high resolution image (d) SAED.

TEM investigations were performed in order to determine the morphology and crystalline nature of the CdMoO_4_ prismatic microsphere sample and the results are shown in Fig. 5a–d. The microspheres can be seen in the TEM image in Fig. 5a. The corresponding TEM images of the CdMoO_4_ microspheres show an ordered uniform structure. A nano prism is observed at the edge of a CdMoO_4_ microsphere, which clearly reveals that the microspheres are composed of nano prisms (Fig. 5b and c). The measured size of the prism is 50–100 nm (Fig. 5c), which is also in good agreement with the results from the FESEM analysis. Fig. 5d shows the selected area electron diffraction (SAED) pattern of the CdMoO_4_ sample, which clearly shows the crystalline nature of the sample.

**Fig. 6:**
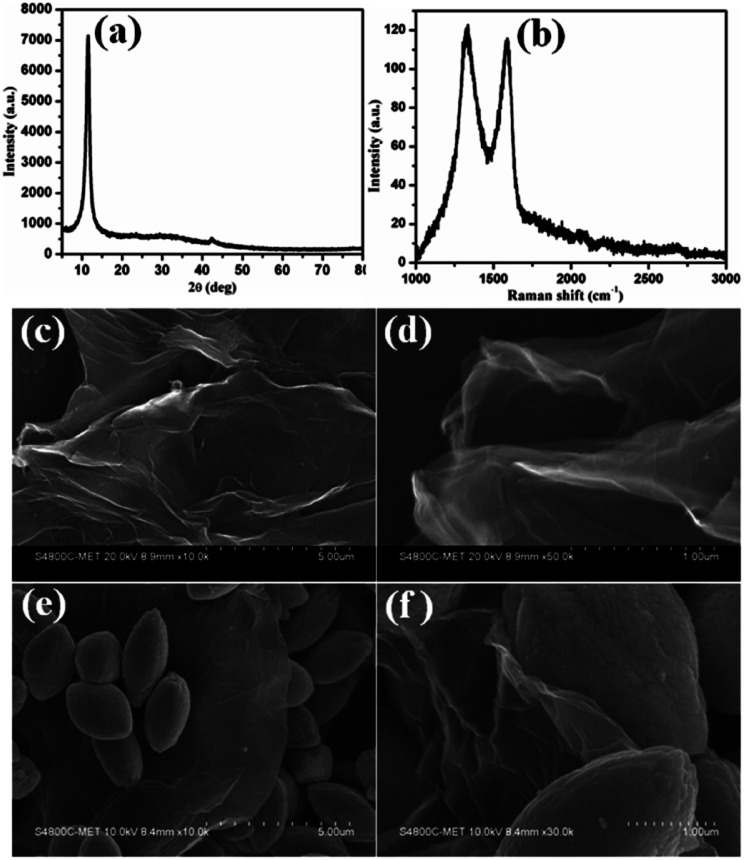
(a) The PXRD pattern of as-synthesized graphene oxide, (b) the Raman spectrum of graphene oxide, (c and d) FESEM images of graphene oxide and (e and f) FESEM images of the CdMoO_4_ nanowire/graphene composite materials.

GO nanosheets were synthesized using an improved Hummers' method. The structural phase formation of the as-prepared GO was investigated using powder X-ray diffraction (PXRD) and the powder pattern is shown in Fig. 6a. Fig. 6a shows an intense peak at 2*θ* = 10.8° and small peaks at 2*θ* = 21.8° and 42.3°, which indicates the phase formation of GO and is in good agreement with the data in the literature.

Raman spectroscopy is a very precise technique that is used to investigate the number of layers present in materials, so it was used to characterize the as-synthesized GO sample and the results are shown in Fig. 6b. The Raman spectrum of GO shows a D band at 1335 cm^−1^ and a broad G band at 1590 cm^−1^, assigned to the first-order scattering of the E_2g_ mode. The prominent D band peak is due to structural defects created by hydroxyl and epoxide functional groups on the carbon basal plane. The surface morphology of the GO layered sheets was investigated using FESEM and the results are shown in Fig. 6c and d. The results confirm the formation of curved sheets of GO with a smooth surface, of various sizes (Fig. 6c and d). The thickness of the observed sheets is approximately ∼5 nm. Fig. 6e and f shows the FESEM images of the CdMoO_4_ nanowire/graphene composite materials. It shows the presence of sheets of graphene decorated with CdMoO_4_ nanowire bundles (5–7 μm size). It can be clearly observed that the nanowire CdMoO_4_ bundles are well dispersed over the multi-layer graphene sheets and remain firmly adhered to them, as is shown in Fig. 6(e and f). This intimate interaction is important for facilitating the transfer of photogenerated electrons from the CdMoO_4_ nanowire bundles to the graphene sheets during the photoexcitation process, which minimises the recombination of electrons (e^−^) and holes (h^+^). The presence of the CdMoO_4_ nanowire bundles anchored on the surface of the graphene sheets results in a larger surface area for moving electrons and holes and makes the material available for photocatalytic activity for a longer time period. This results in more active adsorption sites and photocatalytic reaction centres becoming available, therefore resulting in an enhanced photocatalytic activity.

**Fig. 7:**
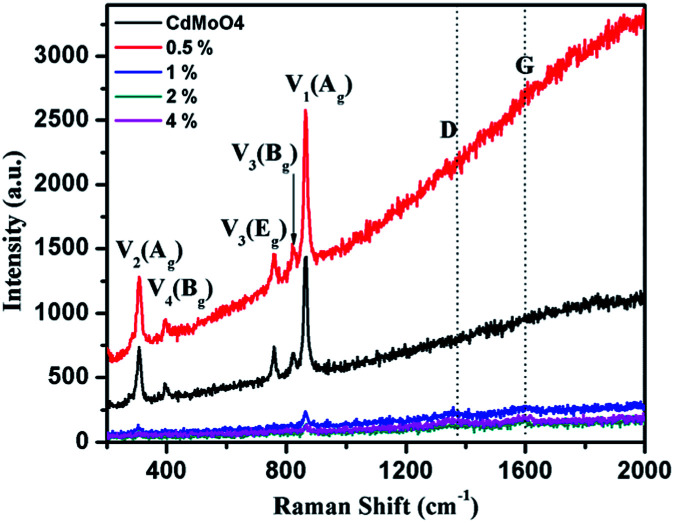
Raman spectra of the as-synthesized CdMoO_4_ nanowires and their 0.5, 1, 2 and 4 weight% graphene composites.

The solvothermally synthesized CdMoO_4_ nanowires and their 0.5, 1, 2 and 4 weight% graphene composites were further analyzed using Raman spectroscopy and the results are shown in Fig. 7a and b. The Raman spectra of CdMoO_4_ and its graphene composites show a peak at 866.1 cm^−1^, assigned as the *ν*_1_(A_g_) symmetric stretching vibration mode of the [MoO_4_] cluster in the CdMoO_4_ structure. The Raman peaks at 820 and 760.5 cm^−1^ can be assigned to the significant anti-symmetric stretching *ν*_3_(B_g_) and *ν*_3_(E_g_) vibration modes of the CdMoO_4_ structure, respectively. Furthermore, the peaks at 397 and 306 cm^−1^ were assigned to the weaker *ν*_4_(B_g_) and stronger *ν*_2_(A_g_) modes of the [MoO_4_] tetrahedrons. A significant D band peak of graphene at 1358 cm^−1^ and a G band peak at 1593 cm^−1^ indicates the presence of layered structured graphene in the composite material. With an increase in the concentration of graphene in the CdMoO_4_ nanowire composites, the Raman peak intensity of the CdMoO_4_ peaks slightly decreases, whereas the magnified D and G band spectra, as shown in Fig. S3 in the ESI,† confirm the presence of graphene in the CdMoO_4_ composite materials.

**Fig. 8:**
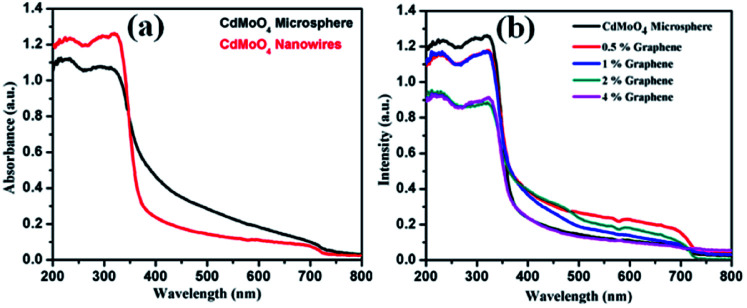
(a) UV-Visible absorption spectra of CdMoO_4_ nanowires and CdMoO_4_ microspheres and (b) UV-Visible absorption spectra of CdMoO_4_ nanowire composites with 0.5%, 1%, 2% and 4% graphene.

The optical properties of the synthesized CdMoO_4_ microspheres, CdMoO_4_ nanowires and CdMoO_4_ nanowire/graphene composites were further investigated using UV-Vis spectroscopy and the results are shown in Fig. 8a and b. It is observed that both the CdMoO_4_ microspheres and nanowires show an absorption edge cut off at ∼375 nm (3.31 eV), as shown in Fig. 8a. It can be clearly observed that the CdMoO_4_ nanowires show a slightly higher absorption than that of the CdMoO_4_ microspheres, which can be attributed to the crystalline nature of the CdMoO_4_ nanowires. In the case of the CdMoO_4_ nanowire composites with graphene, a slight red-shift in the UV-Visible absorption spectra is observed. The absorption edge cut off of the composite materials is observed to be ∼380 nm (3.2 eV) for the 0.5 and 1% graphene composite materials. With an increase in the graphene concentration (in the 2–4% samples), a shift in the absorption spectra towards a longer wavelength at 401 nm (3.1 eV) is observed. The corresponding band gap energy of the composite materials was calculated from the Tauc plot, as shown in Fig. S1 in the ESI.†

The smaller size and crystalline nature of the CdMoO_4_ nanowires allows generated electrons and holes to move easily to the surface of the catalyst, making it available for photocatalytic activity, whereas in the case of the CdMoO_4_ microspheres, the generated electrons and holes cannot move easily to the surface of the catalyst due to the closely packed structure. It is proposed that in the graphene composites, the generated electrons and holes track into the layered graphene and move in the layered structure for a longer time, which reduces the electron and hole pair recombination and makes the material available for photocatalytic activity for a longer time period.

### 3.1 Photocatalytic investigations

The photocatalytic activity of the CdMoO_4_ microspheres, nanowires and the graphene composite materials were evaluated by measuring the amount of H_2_ generated *via* photocatalytic water splitting. The photocatalytic H_2_ generation activity of the synthesized materials was carried out in the presence of solar light. In the present study, methanol was used as a sacrificial reagent. F. Guzman *et al.* earlier discussed the use of methanol as a sacrificial reagent and its detailed mechanism.^47^ It seems that the presence of methanol decreases the e^−^ and h^+^ pair recombination time. Methanol suppresses the evolution of oxygen through the formation of free radicals. As the reactor containing the photocatalyst is irradiated with solar light, the electrons in the conduction band (CB) and holes in the valence band (VB) are generated. The photogenerated holes (h^+^) in the VB oxidize the water molecules into H^+^ ions, while the electrons (e^−^) in the CB reduce the two H^+^ ions into one molecule of H_2_. The photocatalytic water splitting mechanism can be described as follows:Semiconductor catalyst + *hν* → e^−^_(CB)_ + h^+^_(VB)_H_2_O + 2h^+^ → 1/2O_2_ +2H^+^2H^+^ + 2e^−^ → H_2_

**Fig. 9:**
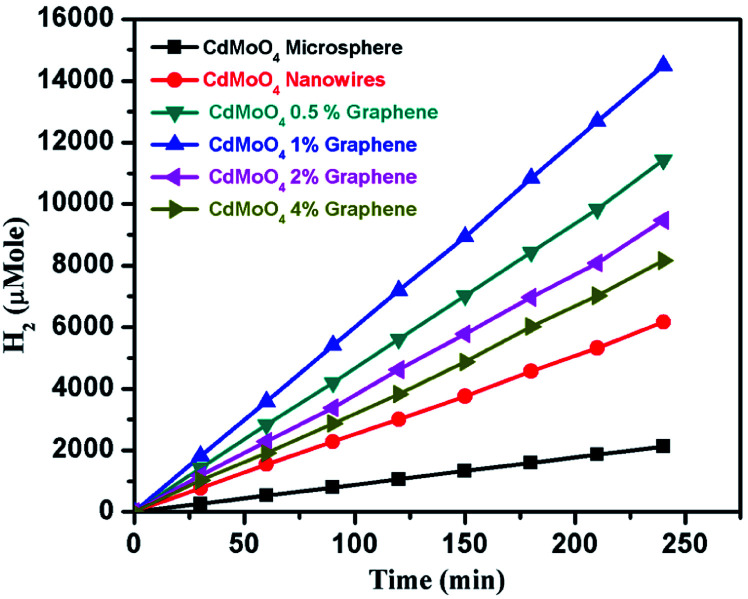
Photocatalytic hydrogen generation activity plot with time for the CdMoO_4_ microspheres, nanowires and the 0.5, 1, 2 and 4 weight% graphene composites.

The results of the amount of H_2_ generated in μmole with respect to time for the CdMoO_4_ prismatic microspheres, nanowires and the graphene composites are shown in Fig. 9 and Table 1. 1540 and 531 μmole h^−1^ g^−1^ of H_2_ were generated for the CdMoO_4_ nanowires and prismatic microspheres, respectively. The linear plots of H_2_ generated in μmole *vs.* the time in minutes are shown in Fig. 9, revealing the continuous H_2_ generation from the water splitting over time. It was concluded that the higher photocatalytic activity observed for the CdMoO_4_ nanowires is due to the small size of the CdMoO_4_ particles, resulting in a greater surface area and increase in the number of active sites for photocatalytic activity. Slightly less hydrogen was generated by the CdMoO_4_ prismatic microspheres than that by the nanowires. The reason for the lower photocatalytic activity is that the nanoparticles in the CdMoO_4_ prismatic structure are agglomerated and closely packed, reducing the surface area and number of active sites, which restricts the easy movement of generated electrons and holes to the surface. It is well acknowledged that the surface morphology and size of the material plays a crucial and significant role in the context of photocatalytic H_2_ generation.^3^ The highest H_2_ generation of 3624 μmole h^−1^ g^−1^ was achieved for the 1% graphene–CdMoO_4_ nanowire composite. It was observed that the H_2_ generation activity increased for the 0.5 and 1% graphene CdMoO_4_ composites. Furthermore, the H_2_ generation activity was observed to decrease for the 2% and 4% graphene composites.

**Table 1:** Photocatalytic hydrogen evolution from water splitting

Sr. no.	Catalyst Name	Graphene (%)	H_2_ Production (in μMol h^−1^ g^−1^)
1	CdMoO_4_ microspheres	0	531
2	CdMoO_4_ nanowires	0	1540
3	CdMoO_4_ nanowires	0.5	2856
4	CdMoO_4_ nanowires	1	3624
5	CdMoO_4_ nanowires	2	2368
6	CdMoO_4_ nanowires	4	2040

**Scheme 1:**
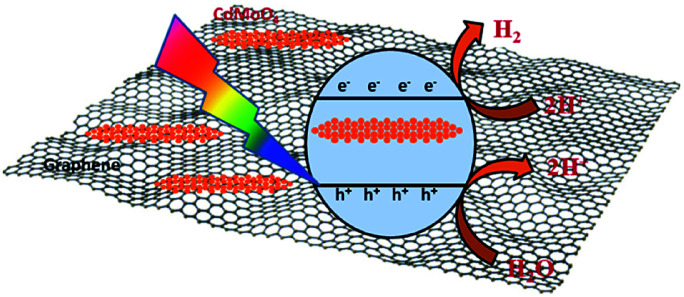
Schematic representation of the photocatalytic hydrogen generation mechanism of the CdMoO_4_–graphene composite photocatalysts.

Interestingly, the 1% graphene/CdMoO_4_ nanowire composite shows almost double the hydrogen generation than that of the pristine CdMoO_4_ nanowires. It can be concluded that the highest photocatalytic H_2_ generation performance of the 1% graphene composite is due to the nanoparticles of the CdMoO_4_ nanowires and edge sites of the graphene and the vacancy defects in the structure. Graphene, in its composite form with semiconductor materials, has exhibited comparatively good photocatalytic activity compared with individual semiconductors due to its highly outstanding properties, such as high electron mobility, large surface area, excellent conductivity, stability and mechanical strength. The 2D layered conjugated structure of graphene in the composite helps to reduce the electron and hole pair recombination time, which is beneficial for the photocatalytic activity. During the photocatalysis experiments, the carbon-related material acted as both an electron-acceptor and electron-transport material; facilitating the migration of photoinduced electrons and obstructing the charge recombination, resulting in an enhanced photocatalytic performance. A schematic representation of the photocatalytic hydrogen generation catalyzed by the CdMoO_4_–graphene composite photocatalysts in natural sunlight is illustrated in Scheme 1.

The small CdMoO_4_ particles facilitate the easy transport of the generated electrons and holes to the surface of the catalyst and track them into the graphene, which reduces the recombination time. Furthermore, there are more defects in the 1% graphene–CdMoO_4_ nanowire/graphene composite, which significantly suppress the recombination of photogenerated charge carriers. Considering the optical properties of the CdMoO_4_ and its graphene composite materials, the higher photocatalytic activity observed for the CdMoO_4_ nanowire/1% graphene composite is quite understandable. As the graphene% increases in the CdMoO_4_ nanowires, an increase in the absorption of visible light is observed. This enhanced absorbance leads to the formation of a large number of e^−^ and h^+^ pairs, resulting in higher photocatalytic activity. In the present study, few layered graphene nanosheets with CdMoO_4_ nanowires were used, which have good stability along with a short carrier diffusion time and hence show good photocatalytic activity. A further increase in the graphene content (in the 2% and 4% graphene composites) led to a decrease in the observed H_2_ generation activity. It is quite reasonable that the introduction of a greater percentage of graphene led to a shielding of the active sites on the catalyst surface. This shielding effect, brought about by the presence of an excess amount of carbon based material in metal oxide catalyst materials has been discussed in detail by Yu *et al.*^48^ Optimized graphene content plays a significant role in attaining the highest photocatalytic activity in the graphene/CdMoO_4_ nanowire composites. In the present study, the highest photocatalytic activity obtained for the 1% graphene CdMoO_4_ nanowire composite is higher than that previously reported for a metal oxide/graphene composite material.^40,49–51^ Overall, the results show that the 1% graphene/CdMoO_4_ nanowire composite is an excellent stable photocatalyst for H_2_ generation in solar light.

This is the only report which reveals photocatalytic H_2_ production *via* H_2_O splitting under solar light catalyzed by a graphene/CdMoO_4_ nanowire composite. The stability of the catalyst was confirmed by performing repeatability studies, which showed that the catalyst retains its activity after being recycled five times, which shows the reproducibility of the results. The PXRD pattern of the reused catalyst sample did not show any change in the phase of the CdMoO_4_ nanowire/graphene composite catalyst, which clearly shows the stability of the catalyst. No H_2_ production was observed in the absence of a catalyst or under dark conditions (in the absence of light). Overall, it is concluded that the CdMoO_4_ nanowire/graphene composite catalyst is quite a stable and active photocatalyst for photocatalytic H_2_O splitting in solar light.

## 4. Conclusion

In conclusion, an *in situ* one step solvothermal approach has been demonstrated for the synthesis of CdMoO_4_ and its graphene composites. The syntheses of CdMoO_4_ nanowires and prismatic microspheres were demonstrated. CdMoO_4_ nanowire bundles were observed to uniformly decorate few layered graphene sheets. Photocatalytic H_2_ generation *via* water splitting in solar light was investigated for CdMoO_4_ and its graphene composites. The highest H_2_ generation of 3624 μmole h^−1^ g^−1^ achieved for the 1% graphene CdMoO_4_ nanowire composite, which has not previously been reported. The small particles, edge sites and defects of the graphene in the catalyst structures play an important key role in the enhancement of H_2_ generation. The generated electrons and holes easily transfer to the surface and quite easily track into the graphene due to the thinness of the graphene nanosheets, which inhibits charge recombination.

## Conflicts of interest

Author has no conflicts of interest.

## Supplementary Material

RA-008-C8RA01557K-s001

## References

[cit1] Luo Z., Miao R., Huan T. D., Mosa I. M., Poyraz A. S., Zhong W., Cloud J. E., Kriz D. A., Thanneeru S., He J., Zhang Y., Ramprasad R., Suib S. L. C. (2016). Adv. Energy Mater..

[cit2] Penner S. S. (2006). Energy.

[cit3] Kadam S. R., Late D. J., Panmand R. P., Kulkarni M. V., Nikam L. K., Gosavi S. W., Park C. J., Kale B. B. (2015). J. Mater. Chem. A.

[cit4] Vaishnav J. K., Arbuj S. S., Rane S. B., Amalnerkar D. P. (2014). RSC Adv..

[cit5] Kadam S. R., Mate V. R., Panmand R. P., Nikam L. K., Kulkarni M. V., Sonawane R. S., Kale B. B. (2014). RSC Adv..

[cit6] Lou S. N., Scott J., Iwase A., Amal R., Ng Y. H. (2016). J. Mater. Chem. A.

[cit7] Turner J. A. (2004). Science.

[cit8] Lubitz W., Tumas W. (2007). Chem. Rev..

[cit9] Apte S. K., Garaje S. N., Arbuj S. S., Kale B. B., Baeg J. O., Mulik U. P., Naik S. D., Amalnerkar D. P., Gosavi S. W. (2011). J. Mater. Chem..

[cit10] Fujishima A., Honda K. (1972). Nature.

[cit11] Maeda K., Domen K. (2010). J. Phys. Chem. Lett..

[cit12] Vequizo J. J. M., Matsunaga H., Ishiku T., Kamimura S., Ohno T., Yamakata A. (2017). ACS Catal..

[cit13] Sheridan M. V., Hill D. J., Sherman B. D., Wang D., Marquard S. L., Wee K.-R., Cahoon J. F., Meyer T. J. (2017). Nano Lett..

[cit14] Roy A., Arbuj S., Waghadkar Y., Shinde M., Umarji G., Rane S., Patil K., Gosavi S., Chauhan R. (2017). J. Solid State Electrochem..

[cit15] Niu M., Huang F., Cui L., Huang P., Yu Y., Wang Y. (2010). ACS Nano.

[cit16] Pawar M., Kadam S., Late D. J. (2017). ChemistrySelect.

[cit17] Baek J. H., Kim B. J., Han G. S., Hwang S. W., Kim D. R., Cho I. S., Jung H. S. (2017). ACS Appl. Mater. Interfaces.

[cit18] Ghodsi V., Jin S., Byers J. C., Pan Y., Radovanovic P. V. (2017). J. Phys. Chem. C.

[cit19] Kadam S. R., Panmand R. P., Sonawane R. S., Gosavi S. W., Kale B. B. (2015). RSC Adv..

[cit20] Na Phattalung S., Limpijumnong S., Yu J. (2017). Appl. Catal., B.

[cit21] Khore S. K., Tellabati N. V., Apte S. K., Naik S. D., Ojha P., Kale B. B., Sonawane R. S. (2017). RSC Adv..

[cit22] Wang J., Waters J. L., Kung P., Kim S. M., Kelly J. T., McNamara L. E., Hammer N. I., Pemberton B. C., Schmehl R. H., Gupta A., Pan S. (2017). ACS Appl. Mater. Interfaces.

[cit23] Sharma R., Khanuja M., Sharma S. N., Sinha O. P. (2017). Int. J. Hydrogen Energy.

[cit24] Kale B. B., Baeg J. O., Lee S. M., Chang H., Moon S. J., Lee C. W. (2006). Adv. Funct. Mater..

[cit25] Xiang Q., Yu J., Jaroniec M. (2012). J. Am. Chem. Soc..

[cit26] Pesci F. M., Sokolikova M. S., Grotta C., Sherrell P. C., Reale F., Sharda K., Ni N., Palczynski P., Mattevi C. (2017). ACS Catal..

[cit27] Zhao W., Ma W., Chen C., Zhao J., Shuai Z. (2004). J. Am. Chem. Soc..

[cit28] Bate N., Shi H., Chen L., Wang J., Xu S., Chen W., Li J., Wang E.-B. (2017). Chem.–Asian J..

[cit29] Shi Y., Wang Y., Wong J. I., Tan A. Y. S., Hsu C.-L., Li L.-J., Lu Y.-C., Yang H. Y. (2013). Sci. Rep..

[cit30] Zhou L., Wang W., Xu H., Sun S. (2008). Cryst. Growth Des..

[cit31] Li D., Zhu Y. (2012). CrystEngComm.

[cit32] Zhen L., Wang W. S., Xu C. Y., Shao W. Z., Ye M. M., Chen Z. L. (2008). Scr. Mater..

[cit33] Xu J., Wu M., Chen M., Wang Z. (2015). Powder Technol..

[cit34] Adhikari R., Malla S., Gyawali G., Sekino T., Lee S. W. (2013). Mater. Res. Bull..

[cit35] Nguyen M., Tran P. D., Pramana S. S., Lee R. L., Batabyal S. K., Mathews N., Wong L. H., Graetzel M. (2013). Nanoscale.

[cit36] Upadhyay R. K., Soin N., Roy S. S. (2014). RSC Adv..

[cit37] Buron J. D., Pizzocchero F., Jepsen P. U., Petersen D. H., Caridad J. M., Jessen B. S., Booth T. J., Bøggild P. (2015). Sci. Rep..

[cit38] Balandin A. A., Ghosh S., Bao W., Calizo I., Teweldebrhan D., Miao F., Lau C. N. (2008). Nano Lett..

[cit39] Qin J., Zhang X., Yang C., Cao M., Ma M., Liu R. (2017). Appl. Surf. Sci..

[cit40] Bhirud A. P., Sathaye S. D., Waichal R. P., Ambekar J. D., Park C.-J., Kale B. B. (2015). Nanoscale.

[cit41] Karthik R., Vinoth Kumar J., Chen S.-M., Karuppiah C., Cheng Y.-H., Muthuraj V. (2017). ACS Appl. Mater. Interfaces.

[cit42] Kumar D. P., Hong S., Reddy D. A., Kim T. K. (2017). Appl. Catal., B.

[cit43] Fatima S., Ali S. I., Iqbal M. Z., Rizwan S. (2017). RSC Adv..

[cit44] Rostami M. (2017). RSC Adv..

[cit45] Wang H., Liang Y., Liu L., Hu J., Cui W. (2017). Appl. Surf. Sci..

[cit46] Marcano D. C., Kosynkin D. V., Berlin J. M., Sinitskii A., Sun Z., Slesarev A., Alemany L. B., Lu W., Tour J. M. (2010). ACS Nano.

[cit47] Guzman F., Chuang S. S. C., Yang C. (2013). Ind. Eng. Chem. Res..

[cit48] Yu J., Ma T., Liu S. (2011). Phys. Chem. Chem. Phys..

[cit49] Shen J., Shi M., Yan B., Ma H., Li N., Ye M. (2011). Nano Res..

[cit50] Li N., Liu G., Zhen C., Li F., Zhang L., Cheng H.-M. (2011). Adv. Funct. Mater..

[cit51] Fan W., Lai Q., Zhang Q., Wang Y. (2011). J. Phys. Chem. C.

