# Supplementary material for: Hierarchical CdMoO4 nanowire–graphene composite for photocatalytic hydrogen generation under natural sunlight
Source: RSC Adv. 2018 Apr 12;8(25):13764–71. doi: 10.1039/c8ra01557k (PMC9079877; doi:10.1039/c8ra01557k)
Supplement: RA-008-C8RA01557K-s001 [file RA-008-C8RA01557K-s001.pdf]

Electronic Supplementary Material (ESI) for RSC Advances.
This journal is © The Royal Society of Chemistry 2018

# Hierarchical $CdMoO_4$ Nanowire-Graphene composite for Photocatalytic Hydrogen Generation under Natural Sunlight

Sunil R. Kadam,[1,2] Rajendra P. Panmand,[2] Shashikant Tekale,[2] Supriya Khore,[2] Chiaki Terashima,[3] Suresh W. Gosavi,[1] Akira Fujishima,[3] Bharat B. Kale[2*]

*

[1]*Centrefor Advanced Studies in Materials Science, Department of Physics, Savitribai Phule Pune University, (Formerly University of Pune)Ganeshkhind, Pune -411007, INDIA*

[2]*Centre for Materials for Electronics Technology (C-MET), Ministry of Electronics and Information Technology (MeitY), Government of India, Panchawati, Off.Pashan Road, Pune –411008, INDIA*

[3]*Photocatalysis International Research Center, Research Institute for Science & Technology, Tokyo University of Science, 2641 Yamazaki, Noda, Chiba 278-8510, JAPAN*

[*]Corresponding author: bbkale@cmet.gov.in,

# Supporting Information

**Experimental Work:**

**A) Synthesis of ($CdMoO_4$ Nanowires)**

The chemicals used for the reaction e.g Cadmium nitrate and Ammonium Molybdate all Qualigens make were used without any purification. The solvent system used for $CdMoO_4$ nano wire synthesis is methanol: ethylene glycol in 3:1 ratio and denoted as a MEG (3:1). Cadmium nitrate dissolved in methanol (120 ml) and Ammonium molybdate were dissolved in ethylene glycol (30 ml ) separately. Ammonium molybdates solution was added drop wise into Cadmium nitrate solution at room temperature with constant stirring. The clear solution of cadmium nitrate turns into little turbid after the addition of the Ammonium molybdates to it. Followed by allow the solution to 10 more minutes to stir with drop wise addition of hydrazine hydrate. The solution becomes faint yellowish coloured. After complete addition mixture was stirred for more 10 min. The reaction mixture is then transferred in teflon lined reactor, packed it in steel jacket. Then the reactor was kept in oven at 150 $^oC$ for 24 hours. After completion of the reaction, the reactor was allowed to cool naturally. The product is filtered by using whatman filter paper no. 41. The product is dried at 80 $^oC$ for 4 hours in oven. The collected powdered sample further analyzed by various characterization techniques.

**B) Synthesis of $CdMoO_4$ prismatic microstructures**

The synthesis of $CdMoO_4$ having Burger like morphology via insitu assembling of prismatic nano particle was synthesized at identical condition like synthesis of $CdMoO_4$ nano wires except solvent system. The solvent system used for the $CdMoO_4$ having Burger like morphology is methanol and ethylene glycol in 1:1 ratio and it is denoted as a MEG (1:1).

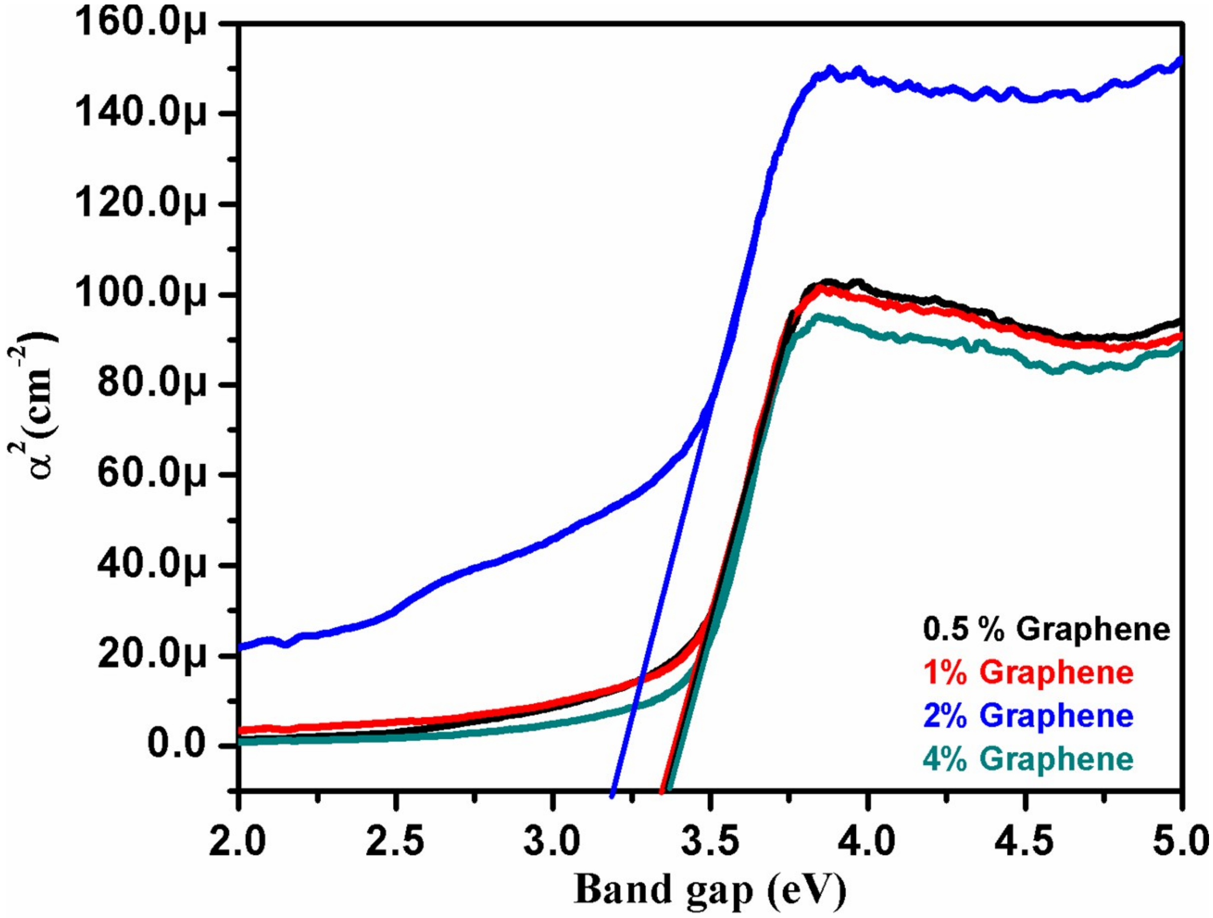


Figure. S1 Tauc plot of CdMoO4 graphene composite materials.

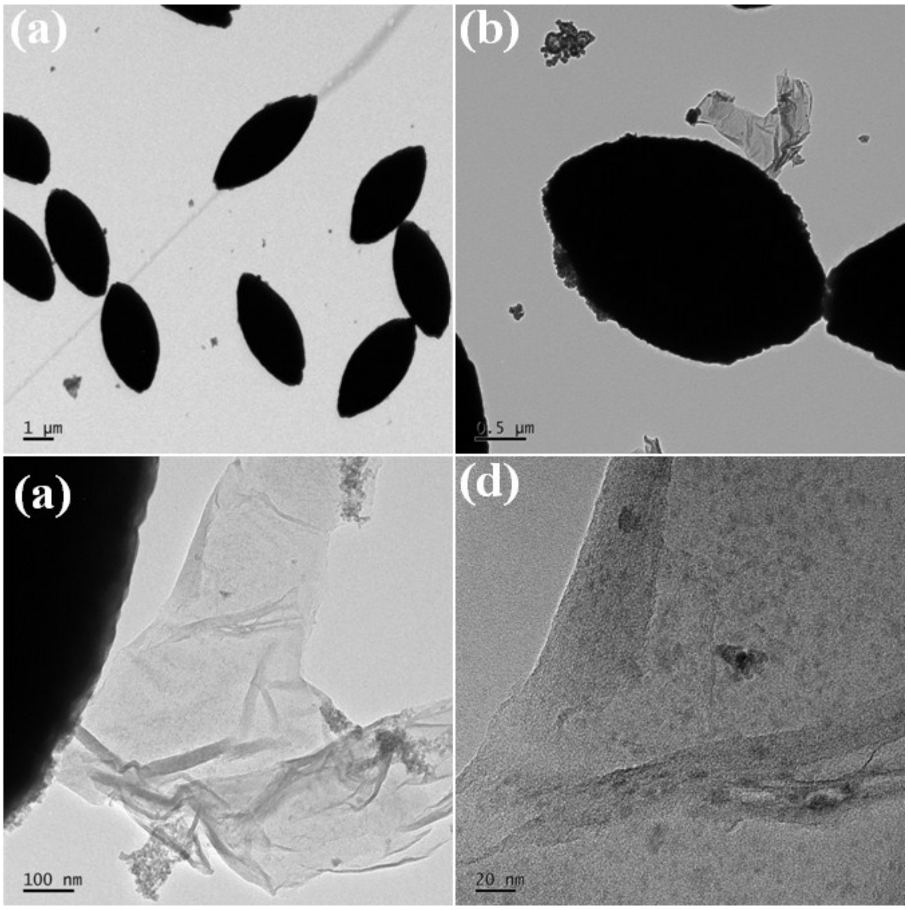


Figure. S2 (a-d) TEM images of as synthesized CdMoO4 nanowires-Graphene

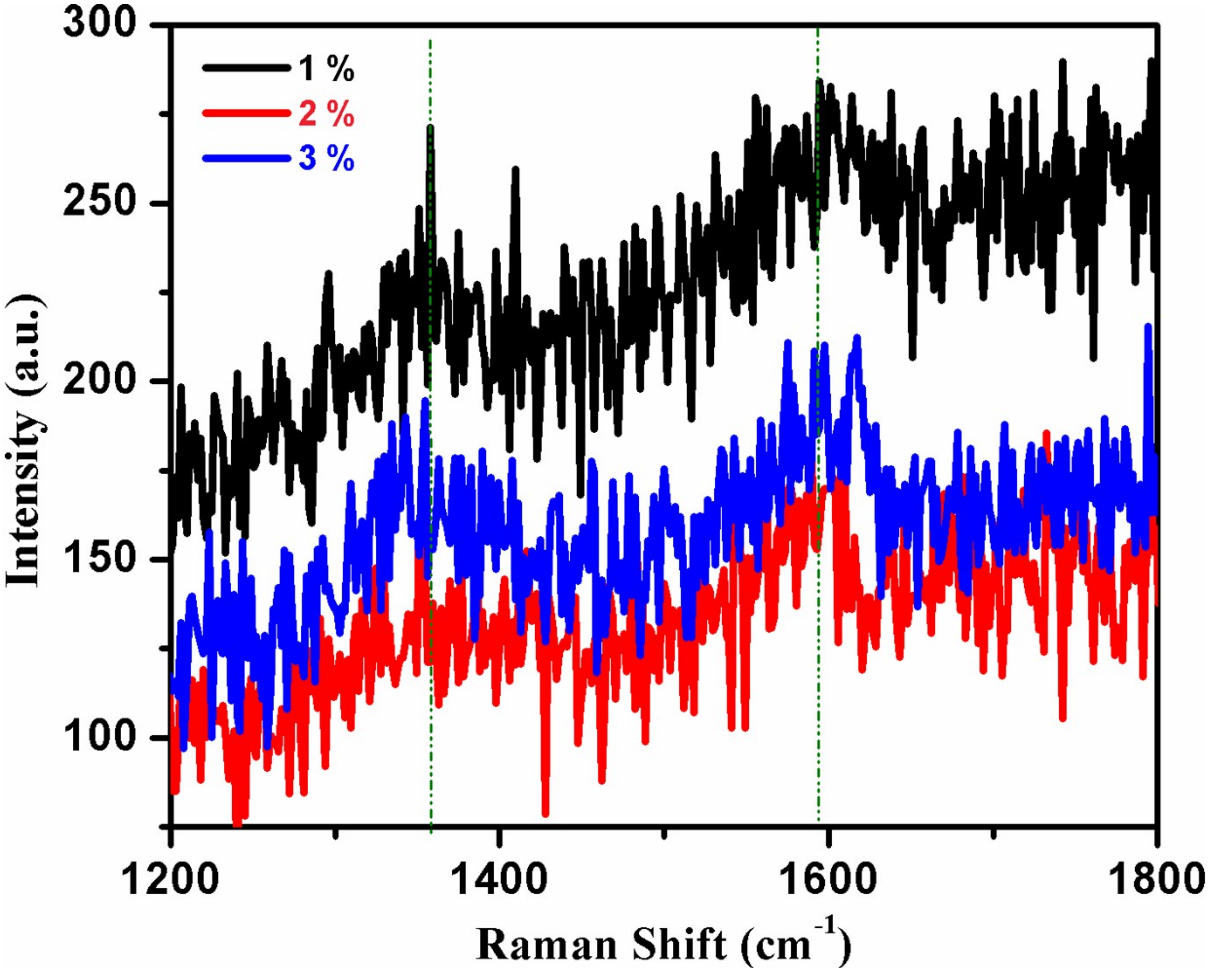


Figure. S3 The magnified Raman spectra for D and G band of $CdMoO_4$ nanowires and its 1, 2 and 4 weight % graphene composite.
